# Recent Advances in Hydrogel-Based Drug Delivery for Melanoma Cancer Therapy: A Mini Review

**DOI:** 10.1155/2017/7275985

**Published:** 2017-08-09

**Authors:** Sowmya Vishnubhakthula, Ravinder Elupula, Esteban F. Durán-Lara

**Affiliations:** ^1^St. Peters Institute of Pharmaceutical Sciences, Kakatiya University, Warangal, Telangana, India; ^2^Department of Chemistry, Tulane University, New Orleans, LA, USA; ^3^Laboratory of Nanobiomaterials, Institute of Chemistry and Natural Resources and Núcleo Científico Multidisciplinario, Universidad de Talca, Talca, Maule, Chile

## Abstract

The purpose of this study is to describe some of the latest advances in using hydrogels for cancer melanoma therapy. Hydrogel formulations of polymeric material from natural or synthetic sources combined with therapeutic agents have gained great attention in the recent years for treating various maladies. These formulations can be categorized according to the strategies that induce cancer cell death in melanoma. First of all, we should note that these formulations can only play a supporting role that releases bioactive agents against cancer cells rather than the main role. This strategy involves delivering the drug via transdermal pathways, resulting in the death of cancerous cells. Another strategy utilizes magnetic gel composites to combat melanoma via hyperthermia therapy. This review discusses both transdermal and hyperthermia therapies and the recent advances that have occurred in the field.

## 1. Introduction

According to the recent statistics from the Skin Cancer Foundation, one in six Americans will develop some form of skin cancer during their lifetime [1]. These cancers are divided into two main categories: nonmelanoma and melanoma. They are the most common types of cancer in the Caucasian population. Melanoma is a less common type of skin cancer; however, it is the most aggressive and is associated with poor prognosis [2]. Malignant melanoma continues to remain an important health threat, with death often occurring as a result of metastasis [3]. The metastatic stage of this cancer is expressed by increased tumor cell invasion and migration of the cancer to other organs [4].

In the pathogenesis of melanoma, the melanocytic skin tumors include an extensive variety of benign and malignant skin lesions with different clinical, morphological, and genetic profiles [5]. Major advances in the understanding of its molecular pathogenesis include the identification of recurrent mutations and aberrations in key signaling and developmental pathways [6, 7]. Melanoma can be caused by both environmental and genetic factors. Melanoma development is multifactorial, and increased susceptibility is associated with extended periods of sun exposure (UV irradiation), recurrent occurrence of sunburn (which is believed to be the most important environmental risk factor), fair pigmentation, advanced age, family history (i.e., genetics are proven to increase the susceptibility of melanoma twofold), higher numbers of melanocytic nevi, chronic immunosuppression, posttransplant patients, and patients with acquired immunodeficiency syndrome or a prior cancer diagnosis [5, 8].

Although the primary cutaneous melanoma can be managed by surgery, the advanced metastatic melanoma cannot be managed by surgery alone; therefore, it demands better therapeutic methods [9].

In the conventional melanoma chemotherapy treatment, antineoplastic agents have been a principal tool for combating cancer [10], specifically dacarbazine, temozolomide, carmustine, lomustine, vincristine, vinblastine, cisplatin, carboplatin, taxol, docetaxel, and so forth [11, 12]. Immunotherapy has become the most widely used treatment for melanoma. This strategy has been applied to the treatment of melanoma utilizing cytokines such as interleukin (IL), IL-2, IL-5, IL-7, and IL-21, interferon-*α* (INF-*α*), and granulocyte macrophage colony-stimulating factor (GM-CSF). Theses cytokines stimulate the patient's immune system to fight cancer. In general, this strategy was successful in fighting malignant cells. However, the main problem of conventional melanoma chemotherapy treatments is the powerful adverse effects, because the neoplastic agents do not distinguish cancer cells from healthy cells [5, 13]. For instance, some of the side effects of cancer melanoma therapy with chemo- and immunotherapy are anorexia, nausea, fatigue, vomiting, renal toxicity, myelosuppression, abdominal pain, dermatitis, hepatitis, and infection, among others [14, 15]. The side effects triggered during conventional treatment encourage the search for new therapy alternatives against cancer cells. Here, we will depict new alternative treatments for melanoma.

## 2. Drug Delivery Systems Based on Hydrogels

New forms of treatments to attack cancer cells are required while simultaneously decreasing the side effects caused in healthy cells [16, 17]. To avoid side effects, transdermal drug delivery systems appear as a promising alternative strategy to carry antineoplastic agents [18, 19]. There are several advantages from using encapsulated antineoplastic agents, including increased drug solubility, better bioavailability, high stability, controlled drug release, prolonged half-life, selective organs or tissue distribution, and reduction of the total dose required. Together, all the benefits outlined above can help minimize adverse side effects to a dramatic degree [20, 21].

Given the adverse side effects caused by conventional therapies in patients with melanoma, a new field, large carrier-based drug delivery systems, has emerged that fights the cancerous cells while subverting the side effects. These types of drug delivery systems possess drug carriers such as nanoparticles [22], dendrimers [23], cyclodextrins [24], liposomes [25], and hydrogels [26] that carry the bioactive antineoplastic agent inside the core/pocket/scaffold. Among these many types of drug delivery methodologies, the development of hydrogels based on natural and synthetic polymers as the drug carriers has received special attention. These biomaterials present an exciting opportunity for designing new methods of cancer therapy [27].

Hydrogels are three-dimensional, hydrophilic polymeric networks that are capable of absorbing large amounts of water, biological fluids, or molecules [28]. Another important property of hydrogels is the ability to swell and dissolve in water [29]. Hydrogels can be divided into chemical and physical gels depending on the nature of crosslinking [30]. These systems possess unique properties to improve the efficacy of the therapeutic agents and minimize undesirable side effects [31]. For an effective therapy, topical and transdermal application of antineoplastic agents from a drug delivery system can mitigate the side effects while improving the drug's efficacy [32].

## 3. Transdermal and Topical Drug Delivery Systems

A successful dermatological treatment depends on both the active compound and the physicochemical properties of the delivery system. Transdermal and topical delivery systems are biomaterials that carry a specific drug in contact with and through the skin [42]. Transdermal and topical administration is the preferred route for local delivery of therapeutic agents due to its convenience and affordability [43]; this is of particular relevance, when referring to skin cancer. Throughout the past, numerous delivery systems or vehicles for topical use such as powders, aerosols, emulsions, and creams were developed. However, currently, hydrogels have demonstrated numerous advantages compared to conventional forms of therapy. This is due to modifiable/tunable hydrogels, where it is possible to control properties of the hydrogel, including degradation rate, long-time release, and tunable pore size [44]. Due to the tenability of hydrogels based on the aforementioned properties, it is worthwhile to investigate hydrogels thoroughly to find optimum hydrogel formulations with specific properties to treat skin cancer.

Traditionally, paclitaxel (PTX) was administered intravenously to treat skin cancer. However, since PTX could not differentiate between healthy cells and cancer cells, it has created many unwanted side effects, sometimes resulting in the patient's death. Therefore, to minimize the cytotoxicity and reduce side effects, targeted delivery of PTX to the cancerous cells, while not affecting the healthy cells, needs to be developed. Ta et al. have developed an approach where the researchers have encapsulated the drug PTX in hydrogels [45]. However, further efforts are required to carry out studies on the efficacy of these hydrogel formulations in* in vitro* and* in vivo* assays in melanoma.

**Figure 1:**
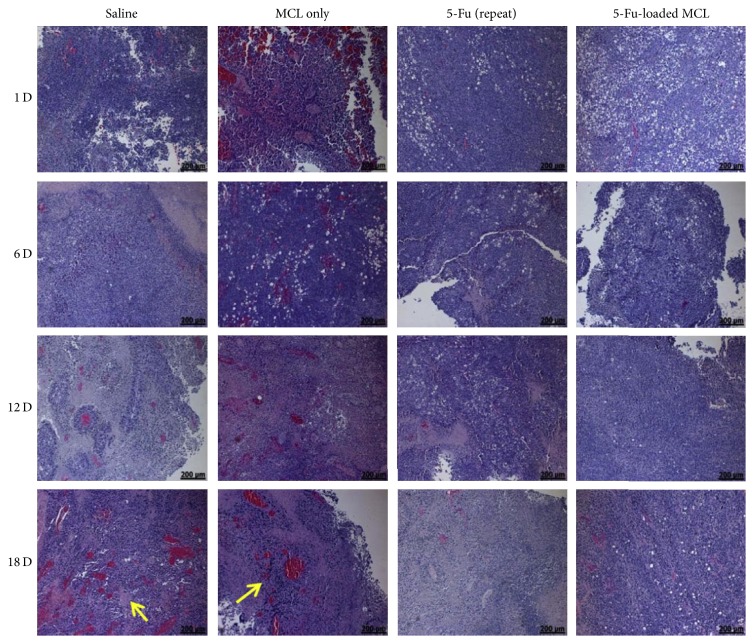
H&E-stained histological sections of tumors on days 1, 6, 12, and 18 after intratumoral injection of saline, MCL only, and free 5-Fu (repeat) and single injection of 5-Fu-loaded MCL in xenograft-bearing mice (arrow indicates the blood vessels, scale bar = 200 *μ*m). Adapted with permission from [33]. Copyright © 2013 Elsevier.

Working on the encapsulation of the bioactive agents in hydrogel scaffolds and subsequent release, Seo and coworkers obtained interesting results. Durable retention of 5-fluorouracil (5-Fu), an antineoplastic agent in a hydrogel based on polyethylene glycol, polycaprolactone, and poly-L-lactic acid copolymers (MCL), within a tumor was demonstrated. Please see Table 1 for the detailed study results. Seo and coworkers revealed that single injection of 5-Fu-loaded MCL was more effective than repeated injections of free 5-Fu. This was confirmed by long-lasting retention of 5-Fu, which induced important inhibition of tumor growth in* in vitro* and* in vivo* assays in melanoma. Furthermore, it was demonstrated that 5-Fu-loaded hydrogel could act as a biodegradable drug depository capable of providing constant release of 5-Fu after intratumoral injection, thus increasing the chemotherapeutic effect of 5-Fu while decreasing its systemic toxicity.  Figure 1 shows hematoxylin and eosin stain, histological sections of tumors inoculated with saline, MCL only, free 5-Fu (repeat), or 5-Fu-loaded MCL. The tumors injected with saline or MCL survived to a large extent and the number of blood vessels (yellow arrow) increased as the implantation time increased. Meanwhile, tumors injected with free 5-Fu (repeat) or 5-Fu-loaded MCL showed an increase in areas containing necrotic tissue that increased over time [33]. These results indicate that it is possible to employ formulations based on hydrogels as a new alternative for drug release platforms. On the other hand, it is vital to evaluate the toxicity of hydrogels and the way they are metabolized, degraded, and removed from the body for avoiding side effects related to this type of formulation.

Since the beginning, classical pharmacology had to adapt to disadvantages of some antineoplastic agents such as low hydrophobicity. In this context, enormous efforts have been made to correct this problem with the usage of hydrogels. For instance, curcumin (Cur) is a hydrophobic polyphenol with antiproliferative and antiangiogenic activities. However, its weak water solubility and photochemical instability have been the major obstacles for its formulation. Cur has been encapsulated into the hydroxypropyl-*β*-cyclodextrin supported* in-situ*-forming hydrogels. The therapeutic effect of* in vitro* Cur on melanoma has been investigated. The solubility, stability under light, erosion, release* in vitro*, transdermal permeability efficiency, and cytotoxicity of Cur inclusion complexes have been improved by encapsulating Cur in hydrogel scaffolds. Therefore, Cur encapsulated in hydrogel is a promising formulation for melanoma treatment. Nevertheless, future studies on the developed formulation are necessary for validating the therapeutic effect in an* in vivo* setting [34].

There is evidence that inflammation stimulates tumor invasion or cancer metastasis [35]. Inflammatory responses play key roles at different stages of tumor development, including initiation, promotion, malignant conversion, invasion, and metastasis [46, 47]. Consequently, in light of all this evidence, some studies have emerged that have investigated the effect of nonsteroidal anti-inflammatory drugs (NSAIDs), such as aspirin, in individuals who were on long-term cancer treatment. Specifically, ibuprofen-releasing hydrogel (Pluronic® F127) has been developed as a new approach of transdermal formulation for reducing metastatic spread of primary melanoma. The results obtained suggested that ibuprofen significantly reduced TNF-a-stimulated migration in* in vitro *melanoma cell lines. Furthermore, the hydrogel and ibuprofen combination has been demonstrated to be capable of inhibiting cell migration [48].

In recent years, studies have been carried out with the goal of achieving efficient release of doxorubicin (DOX) through a formulation based on hydrogel. Some research studies have investigated chemotherapy for melanoma in depth. These studies have utilized a natural protein from silk, sericin, with dextran to form hydrogels and later incorporate DOX to use a drug delivery system. The hydrogel loaded with DOX, subcutaneously injected, significantly suppressed tumor growth in the* in vivo* melanoma model in male mice. Moreover, this hydrogel has proven to be biodegradable and biocompatible [36].

**Scheme 1:**
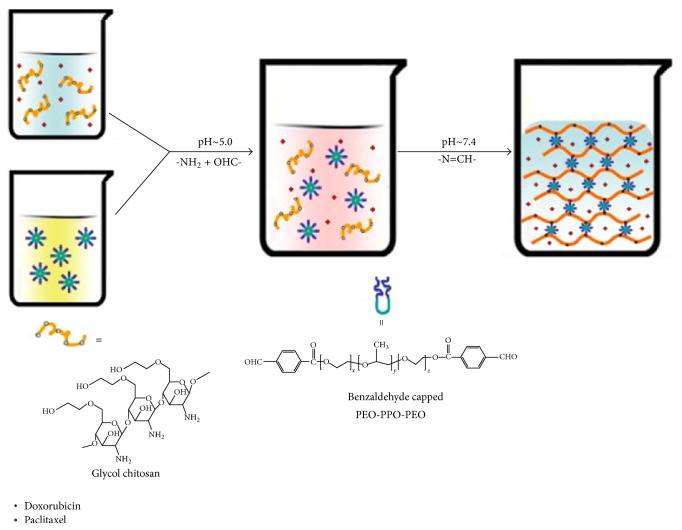
Formulation of injectable GC-OHC-PEO-PPO-PEO-CHO gel containing DOX and PTX. Adapted with permission from [37]. Copyright © 2011 Elsevier.

Also, advances have been made in the combination therapy, where two or more drugs are used to combat cancer. This type of combinatorial system was developed based on an injectable hydrogel of glycol chitosan benzaldehyde capped poly(ethylene glycol)-block-poly(propylene glycol)-block-poly(ethylene glycol) (GC-OHC-PEO-PPO-PEO-CHO). This formulation has been prepared as a flexible vehicle for intratumor delivery of two anticancer drugs with different solubility characteristics, DOX and PTX. The GC-OHC-PEO-PPO-PEO-CHO injectable gel is able to* in situ* contain both drugs due to the inclusion of the amphiphilic macromolecular cross-linker. The absorption procedure is illustrated in Scheme 1. Better results were obtained by Yang et al. where the authors have achieved faster drug release at tumor pH than at physiological pH.* In vivo* assays (murine melanoma cancer) demonstrated the ability of the formulation as a depository to reduce the amount of drug being transferred to the bloodstream, consequently decreasing the toxic effects of the drug. The gel exhibited tumor inhibition efficacy. The efficiency of the system for combined DOX and PTX therapy has been confirmed by significant prolongation of the survival rate of the tumor bearing mice [37].

Tomic's group synthesized and evaluated a series of dual-sensitive poly(2-hydroxypropyl acrylate/itaconic acid) (P(HPA/IA)) hydrogels as a potential highly effective antiproliferative drug delivery system. Ni(II) complex with oxaprozin, a hydrophobic antiproliferative agent, was synthesized and loaded into the P(HPA/IA) hydrogels. The studies showed that loaded antiproliferative agents did not annul pH and temperature sensitivity of the investigated hydrogels.* In vitro* antiproliferative activity of investigated complexes against melanoma cancer cell lines was tested. The results of the* in vitro* release study at different pH values confirmed that the synthesized complex is a highly effective pH-triggered drug delivery system for advanced anticancer therapy [38].

A few more alternatives of drug delivery systems have also been developed. A new injectable biodegradable scaffold for the delivery of living T-lymphocyte cultures was developed. Using combinations of sodium hydrogen carbonate and phosphate buffer as gelling agents, novel injectable chitosan-based biocompatible thermogels (CTGels) have been achieved. They have excellent mechanical characteristics and cytocompatibility. It was determined that T-cells remained cytotoxically functional to kill cancer cells of melanoma* in vitro* after encapsulation into hydrogel based on chitosan [49]. However, this type of drug release has limitations in that if the therapy does not utilize the T-lymphocytes from the same host body, major complications may occur.

Finally, a new anticancer therapy strategy that utilizes enzymes to treat cancer was proposed by Matricardi et al. Authors of this study employed bovine serum amine oxidase (BSAO) to treat cancer. This enzyme converts polyamines that are overexpressed in malignant cells into hydrogen peroxide and aldehyde(s), thus inducing high cytotoxicity in cancer cells. In this regard, to improve delivery efficiency, they developed a formulation where BSAO is covalently immobilized into injectable nanohydrogels (NHs) based on cholesterol-graft-hyaluronic acid (HA-CH), a biocompatible conjugate that immediately leads to self-assembled structures in aqueous solutions [39]. Agostinelli et al. proposed a “new BSAO delivery system” that immobilizes BSAO on other hydrogel polymers, specifically arginate/chitosan. The results of this particular approach were quite promising; however, it needs to be explored in depth to be able to become a viable therapy for human cancer [40].

## 4. Combinatorial Drug Therapy

Owing to the molecular complexity of numerous cancers, combination therapy is becoming increasingly important for a better long-term prognosis and to reduce side effects. Combinatorial drug therapy for the treatment of a disease normally refers to either the simultaneous administration of two or more pharmacological drugs or the combination of different types of therapy, for instance, chemotherapy and radiotherapy [50]. In contrast to single-agent therapy, multiagent therapy can modulate different signaling pathways in diseased cells, maximizing the therapeutic effect and overcoming the mechanisms of resistance [51]. In the context of this article, the hydrogels can readily sequester water-soluble drugs, providing a means for combinatorial therapy against cancer [52]. In this manner, the hydrogel provides an interesting platform to design combination therapy and drug release formulation [53].

## 5. Development of Magnetic Gel Composites for Hyperthermia Therapy

Chemotherapy is most commonly used for the treatment of cancer patients but also develops undesirable side effects. In recent years, innovative treatment approaches such as hyperthermia with gold nanoparticles, magnetic nanoparticles, and the combination of hyperthermia and chemotherapy are being investigated for improving patient's quality of life [54]. Magnetic nanoparticles are increasingly studied and exploited for their potential applications in cancer treatment [55]. Hyperthermia therapy is a medical treatment based on the exposure of body tissue to temperatures slightly higher than the physiological temperature (i.e., between 41°C and 46°C) to damage and kill cancer cells or to make them more susceptible to the effects of radiation and anticancer drugs. Although it still is an experimental method, local hyperthermia has shown to be successful in clinical tests when combined with well-developed chemotherapy or radiotherapy for melanoma cancer [56]. However, more studies are required in this field.

**Figure 2:**
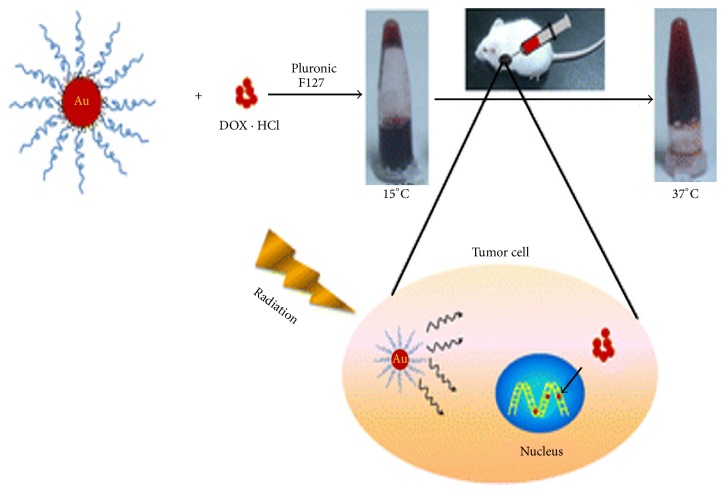
Pluronic F127 hydrogel coloaded with AuNPs and DOX for effective cancer chemoradiotherapy. Adapted with permission from [41]. Copyright © 1999–2017 John Wiley & Sons, Inc.

Li and coworkers carried out studies in melanoma tumor-bearing mice; the result obtained demonstrated that an enhanced cancer chemoradiotherapy was achieved by a Pluronic F127-based thermosensitive hydrogel loading AuNPs and DOX (Au-DOX-Gel), in which DOX acted as a chemotherapeutic while polyethylene glycol (PEG) modified gold nanoparticles that (AuNPs) killed tumor cells by enhancing the radiation dose.  Figure 2 shows the Pluronic F127 hydrogel coloaded with gold nanoparticles (AuNPs) and DOX. The AuNPs and DOX could be coloaded into hydrogels and then injected into the tumor. After radiation, AuNPs would act as a radiosensitizer and DOX would treat the tumor as a chemotherapeutic [41].

On the other hand, studies carried out by Agostinelli et al. corroborated that the toxic enzymatic oxidation products generated by BSAO and polyamines can be used along with hyperthermia (42°C) or with other drugs such as lysosomotropic compounds in the treatment of solid tumors such as melanomas (combinatorial treatment) [40].

The strategies that have been developed in the last years and that are described in this article are summarized in Table 1.

## 6. Conclusion

Developing a drug delivery system for effective cancer therapy where we can track the activity* in vivo* noninvasively is highly desired, mainly with the goal of avoiding or decreasing side effects.

One of the greatest advantages of developing a drug delivery system for a targeted therapy for melanoma skin cancer compared with other cancers is its easier accessibility. Perhaps for this reason there are several advances in this area (drug delivery system using melanoma cancer model). On the other hand, there are several investigations that have designed drug delivery systems with numerous antineoplastic agents that are used to treat different types of cancer, which included breast cancer, colon cancer, and ovarian cancer. The previously mentioned approaches could be tested in melanoma skin cancer as well.

The usage of formulations based on hydrogels in melanoma skin cancer as a delivery system of antiproliferative agents has several advantages over other drug delivery systems and conventional therapies. The polymer engineer can design and synthesize hydrogel with molecular-scale control over structures such as cross-linking density and with tailored properties, such as biodegradation, degradation rate, pore size, mechanical strength, and chemical and biological response to stimuli such as pH, enzymes, and temperature. Another important benefit of hydrogel is the low cost compared with the other polymeric formulations such as nanoparticles, microparticles, and dendrimers. Therefore, the modifiable and highly tunable properties of hydrogel make it a preferred choice in melanoma cancer therapy. In the future, it is necessary to make changes such as concentration of antineoplastic agents applied and stage of cancer. These parameters were not discussed during this review because this is not the purpose of this work.

## Figures and Tables

**Table 1:** Melanoma cancer formulations summarized.

Polymer	Antineoplastic agents/action	Cancer	Route of administration	Reference
MPEG-b-(PCL-ran-PLLA) diblock copolymer (MCL)	5-Fluorouracil	Melanoma *in vivo* mice model tumor	Subcutaneously injected drug delivery system	Seo and coworkers [33]

Hydroxypropyl-*β*-cyclodextrin supported in *in-situ*-forming hydrogels with poloxamers 407 and 188	Curcumin/antioxidant, anti-inflammatory, antiproliferative and antiangiogenic activities	*In vitro* melanoma model in cell lines	Transdermal drug delivery system	Sun and coworkers [34]

Pluronic F127	Ibuprofen/nonsteroidal anti-inflammatory	*In vitro* melanoma model in cell lines	Topical/transdermal drug delivery system	Marques and MacNeil [35]

Hydrazone cross-linked sericin/dextran	Doxorubicin	*In vivo* melanoma model in male mice	Subcutaneously injected drug delivery system	Liu and coworkers [36]

Glycol chitosan benzaldehyde capped poly(ethylene glycol)-block-poly(propylene glycol)-block-poly(ethylene glycol) (GC-OHC-PEO-PPO-PEO-CHO)	Paclitaxel and doxorubicin (combination therapy)	*In vivo* melanoma model in male mice	Subcutaneously injected drug delivery system	Zhao and coworkers [37]

Poly(2-hydroxypropyl acrylate/itaconic acid) (P(HPA/IA))	Ni(II) complex with oxaprozin/antiproliferative activity	*In vitro* melanoma cancer cell lines	Topical/transdermal drug delivery system	Babić and Coworkers [38]

Nanohydrogels (NHs) based on cholesterol-graft-hyaluronic acid	Bovine serum amine oxidase (BSAO)	*In vitro* melanoma cancer cell lines	Topical drug delivery system	Montanari and coworkers [39]

Arginate/chitosan	Bovine serum amine oxidase (BSAO)		Topical drug delivery system	Agostinelli and coworkers [40]

Pluronic F127	AuNPs and DOX	*In vivo* melanoma model in male mouse	Subcutaneously injected drug delivery system/hyperthermia therapy	Li and coworkers [41]
